# Diacylglycerol Signaling Underlies Astrocytic ATP Release

**DOI:** 10.1155/2011/537659

**Published:** 2011-06-13

**Authors:** Alison E. Mungenast

**Affiliations:** ^1^Department of Neuroscience, Tufts University, 136 Harrison Avenue, Boston, MA 02111, USA; ^2^Department of Neuroscience, Perelman School of Medicine at University of Pennsylvania, Philadelphia, PA 19104, USA

## Abstract

Astrocytes have the ability to modulate neuronal excitability and synaptic transmission by the release of gliotransmitters. The importance of ATP released downstream of the activation of Gq-coupled receptors has been well established, but the mechanisms by which this release is regulated are unclear. The current work reveals that the elevation of diacylglycerol (DAG) in astrocytes induces vesicular ATP release. Unexpectedly, DAG-induced ATP release was found to be independent of PKC activation, but dependent upon activation of a C1 domain-containing protein. Astrocytes express the C1 domain-containing protein Munc13-1, which has been implicated in neuronal transmitter release, and RNAi-targeted downregulation of Munc13-1 inhibits astrocytic ATP release. These studies demonstrate that elevations of DAG induce the exocytotic release of ATP in astrocytes, likely via a Munc13-1-dependent mechanism.

## 1. Introduction

The role of astrocyte gliotransmission in the modulation of neuronal signaling has been reported upon with increasing frequency within the last decade [1–3]. Astrocytes have been shown to release gliotransmitters, including glutamate and D-serine, via vesicular exocytosis [2, 4–10], which influence synaptic properties [1, 9]. In addition, astrocytic ATP release plays important roles in synaptic plasticity [1, 11–13] although the cell signaling events preceding its release are currently unclear [14]. 

Astrocytes express class I metabotropic glutamate receptors (mGluRs), which can induce gliotransmission in response to neuronal glutamate [15–17]. Previous work has shown that activation of astrocytic mGluR5 modulates neurotransmission in the nucleus accumbens [18]. mGluR5 receptors are G-protein-coupled receptors (GPCRs) that signal via the Gq and phospholipase C (PLC) signaling pathway, in which the small signaling molecules 1,4,5-trisphosphate (IP_3_) and diacylglycerol (DAG) are produced, and IP_3_-stimulated calcium release is effected from internal stores. The DAG analogue 1-oleoyl-2-acyl-sn-glycerol (OAG) has been found to induce calcium oscillations in astrocytes in a PLC-dependent manner through an undefined pathway [19]. This finding suggests that gliotransmission may be effected via activation of the DAG arm of the Gq signaling pathway, although the mechanism responsible for the observed calcium oscillations is not clear. 

ATP is released from astrocytes in the brain where it is rapidly converted to adenosine in the perisynaptic space through the actions of ectonucleotidases [20]. This adenosine acts to tonically inhibit synaptic transmission through activation of presynaptic A1 receptors [1]. Transgenic animals with impaired SNARE-dependent astrocytic ATP release demonstrate that adenosine, converted from astrocytic ATP, suppresses neuronal excitability and leads to heterosynaptic depression [1, 21]. In addition to serving physiological roles, astrocytes can contribute to excitation underlying seizures [22], although the precise role of gliotransmitters remains to be determined. Adenosine, acting through A1 receptors, causes a reduction in neuronal excitability and in excitatory synaptic transmission [23]. So powerful are these actions that small-molecular-weight adenosine analogs have been proposed as anticonvulsants (for review see [24]). However, their systemic actions limit their effective use as therapeutics. Thus, the elucidation of the intracellular signaling pathways connecting the activation of Gq GPCRs, like mGluR5, to astrocytic ATP gliotransmission provides new targets for the therapeutic manipulation of neuronal excitability. In the current work, I provide evidence that activation of the DAG pathway in astrocytes leads to exocytotic ATP release. 

## 2. Materials and Methods

### 2.1. Reagents

OAG was purchased both in solid form from Biomol (Plymouth Meeting, Pa) and as a solution in acetonitrile from Cayman Chemical (Ann Arbor, Mich). For short-term storage (less than 2 weeks), solid OAG was dissolved in fresh DMSO to 60 mM and stored under nitrogen in screw-top tubes at −80°C. To prepare for use, aliquots in DMSO were thawed at room temperature and sonicated for 5 minutes. The OAG was then added to warm (~37°C–42°C) normal hippocampal saline (NHS; concentrations (in mM) 140 NaCl, 5 KCl, 2 CaCl_2_, 2 MgSO_4_, 10 HEPES, 10 D-glucose, and 6 Sucrose, pH = 7.35) and vortexed well. For longer-term storage (up to 2 months), OAG in acetonitrile was dried under nitrogen in 1 mg aliquots and stored under nitrogen at −80°C. Fresh, warm DMSO was added to make 60 mM OAG, and this solution was sonicated for 5 minutes before being added to warmed NHS as above. Final concentrations of both 30 *μ*M and 100 *μ*M of OAG in NHS were used in these experiments. OAG solutions were maintained at or above room temperature, to maintain the solubility of the OAG.

Other experimental reagents included 1-Stearoyl-2-arachidonoyl-sn-glycerol (SAG, 60 mM stock in DMSO, Biomol), Suramin (stock fresh in H_2_O, Sigma, St. Louis, Min, USA), pyridoxalphosphate-6-azophenyl-2^'^,4^'^-disulphonic acid (PPADS, stock fresh in H_2_O, Tocris, Ellisville, Min), Bafilomycin A1 (100 *μ*M stock in DMSO, Biomol), Carbenoxolone (80 mM stock in H_2_O, Sigma), GF 109203X (Bisindolylmaleimide I, 20 mM in DMSO, BioSource, Camarillo, Calif, USA), Calphostin C (10 mM stock in DMSO, Biomol), ATP (made fresh in NHS, Sigma), Apyrase (50 U/mL stock in H_2_O, Sigma), RHC-80267 (60 mM stock in DMSO, Biomol), R59949 (25 mM stock in DMSO, Calbiochem, San Diego, CA, USA). Nominally calcium-free NHS contained about 60 nM of calcium to prevent calcium efflux from astrocyte internal stores. 

The OAG used in the experiments was confirmed to be free from contamination by ATP using the luciferin-luciferase luminescence reaction. NHS containing 30 *μ*M and 100 *μ*M OAG was combined with Sigma's ATP assay reaction mix (FLAAM) and assayed for luminescence using a Synergy 2 Luminometer from Biotek (Winooski, Vt, USA). An absence of luminescence in the OAG samples confirmed that they were not contaminated with ATP. 

### 2.2. Culture of Primary Cortical Astrocytes

Cortical astrocytes from neonatal C57/Bl6 mice were cultured as described previously in [7, 8]. After 2 weeks in culture, astrocytes were plated onto coverslips coated with 10 *μ*g/mL poly-D-lysine and cultured in regular MMEM for 7 to 21 days before imaging. Prior to imaging, coverslips were washed with normal hippocampal saline and incubated for 40 minutes at room temperature with Fluo-4 (Invitrogen, Carlsbad, CA) in NHS (5 *μ*g/mL Fluo-4 with 1 : 1000 20% Pluronic acid in DMSO). Nominally Ca^2+^-free NHS was composed as follows (concentrations (in mM) 140 NaCl, 5 KCl, 1 CaCl_2_, 2 EGTA, 2.15 MgSO_4_, 10 HEPES, 10 D-glucose, and 6 Sucrose). The final concentration of free external calcium was estimated to be 60 nM. For experiments using Bafilomycin A1, cells were incubated with 100 nM Bafilomycin A1 or the equivalent amount (0.1%) of DMSO for 45 minutes at 37°C and then for an additional 45 minutes at room temperature with the addition of Fluo4. For experiments using Calphostin C, cells were incubated for 15 minutes following *t*1 with 100 nM of Calphostin C under room lights to activate the compound, and lights were then turned off for imaging. 

### 2.3. Imaging

Coverslips were placed on the stage of an epifluorescence microscope equipped with a cooled digital camera (ORCA, Hamamatsu) controlled by Image-Pro software with custom-build function (Phase 3 Imaging Systems, Pa, USA). All experiments were done in NHS with perfusion kept at 33°C with an in-line solution heater. 

Time series experiments consisted of three series of 200 time-lapse images (*t*1, *t*2, and *t*3) separated by 15-minute rinses in NHS. The interval between frames was approximately 5 seconds. Most pharmacological manipulations were added during the *t*2 interval. Image stacks were analyzed using the Metamorph software package. Fluorescence intensities were then measured on image stacks, and data was exported to and analyzed in MS Excel. Measurements shown include *F*/*F*_0_ (increase in fluorescence over baseline) and the percentage of astrocytes in a field with *F*/*F*_0_ > 2 (% cells responding). 

### 2.4. Immunostaining

Immunocytochemistry was performed on astrocytes plated, as above, on coverslips and fixed with 4% paraformaldehyde for 20 minutes at room temperature. Coverslips were incubated in blocking buffer (50 mM NH_4_Cl containing 2% BSA, 2% glycine, and 0.2% gelatin) for 15 minutes at RT. To localize Munc13-1 protein, coverslips were incubated in affinity-purified rabbit polyclonal anti-Munc13-1 (1 : 100, Synaptic Systems) in blocking buffer for 48 hours at 4°C followed by the secondary antibody (goat antirabbit IgG-Alexa 588, 1 : 500, Invitrogen) for 1 hour at room temperature. Coverslips were mounted onto microscope slides with Vectashield Hardset mounting medium with DAPI (Vector Labs). Images were taken at 20x on a Nikon A1 confocal laser microscope and color-combined in Metamorph. 

### 2.5. Western Blotting

Protein used for Western blotting was extracted from confluent astrocyte cultures in 10 cm dishes. Cells were rinsed with PBS and snap-frozen on dry ice; protein was then extracted with Pierce's M-PER extraction buffer (Pierce) containing protease inhibitors (Halt Protease Inhibitor, Fisher) and quantified using the Bio-Rad protein assay. For positive controls, cultured mouse cortical neurons were similarly processed. Sixty micrograms of astrocytic protein or 15 *μ*g of neuronal, was loaded onto each lane of a 7% Tris-Acetate gel (NuPage, Invitrogen). Following electrophoresis, proteins were transferred to Hybond C extra nitrocellulose membranes (Amersham Biosciences), blocked with 4% nonfat milk in TBS buffer containing 0.05% Tween-20 (TBST). Membranes were then cut in half to make a high-MW membrane (containing Munc13-1, 200 kD) and a low-MW membrane (containing GAPDH, 36 kD). The high-MW membrane was incubated overnight at 4°C with rabbit polyclonal anti Mun13-1 (Synaptic Systems) used at 1 : 500 in TBST. The low-MW membrane was incubated overnight with mouse monoclonal anti-GAPDH (Fisher) at 1 : 1000. Membranes were then washed in TBST and incubated for 1 hour in secondary antibody (goat antirabbit-HRP 1 : 5000, goat anti-mouse-HRP Pierce 1 : 3000). Immunoreactive bands were detected by exposing membranes to Amresco VisiGlo HRP-plus chemiluminescent substrate for 5 minutes (Amresco, Solon, OH) and imaging with the LAS 3000 luminescence imaging system (Fuji Film). Quantitative Western blotting was performed by quantifying Munc13-1 optical density as a proportion of GAPDH using ImageJ software (NIH). 

### 2.6. siRNA

Silencer Select predesigned siRNAs against mouse Munc13-1 (NM_0010298873) were obtained from Ambion (Austin, Tex, USA) and resuspended in RNAse-free sterile water to make a 20 *μ*M stock which was stored at −80°C. Primary astrocytes on 10 cm plates or coverslips were transfected with 3.2 *μ*g/mL siRNAs in culture with the TransMessenger transfection reagent (Qiagen, Valencia, CA, USA). Silencer Negative Control no.1 siRNAs and Silencer Cy3-labeled Negative Control no.1 siRNAs, both from Ambion, were used as controls. Examination of cultures transfected with Cy3-labeled siRNAs demonstrated a near 100% transfection efficiency of the siRNAs (data not shown). Seven days after transfection astrocyte protein was collected from the 10 cm plates as described above. Western Blot analysis demonstrated a moderate (30%) reduction of Munc13-1 protein in astrocytes transfected with Munc13-1 siRNAs compared with negative control siRNAs. Five to 7 days after transfection, siRNA-transfected astrocytes on coverslips were imaged for OAG response as described above. 

### 2.7. RT-PCR, Cloning, and Sequencing of Mun13-1

PCR primers were made against a portion of the coding domain of mouse Munc13-1 (GenBank NM_001029873). Five hundred nanograms of RNA extracted from mouse cortical astrocytes with Trizol reagent was subjected to reverse transcription using Qiagen Omniscriot RT (Qiagen, Valencia, CA). With the primers described above, PCR was performed using Qiagen HotStar Taq (Figure 6(c)). The resultant PCR product was cloned into the pGEMT-Easy cloning vector (Promega) and transformed into XL-1 blue competent cells (Stratagene, La Jolla, CA). Blue/white screening on LB/AMP plates determined positive colonies which were maxiprepped and the inserts confirmed by restriction digest. Several inserts were sequenced using M13 forward and reverse primers on an Applied Biosystems (ABI; Foster City, CA) 3130XL sequencer. Resultant sequences were aligned with NM_001029873 using DNAStar MegAlign software (Madison, Wis) and 100% homology was confirmed. 

### 2.8. Luciferase Assays

Astrocytes were subcultured into the inner wells of 96-well Corning white, clear-bottomed assay plates (Corning Inc, Corning, NY, USA) at 16,000 cells/well. Outer wells contained sterile water to prevent uneven evaporation during culturing. Plates were brought to an incubator adjacent to the luminometer (Synergy2, BioTek, Winooski, Vt, USA) at least 24 hours prior to assay to prevent unnecessary motion. Astrocytes were rinsed once with 50 *μ*L of warmed NHS and rested for 40 minutes at room temperature. Following the addition of 60 *μ*M OAG in 50 *μ*L NHS (final concentration = 30 *μ*M), plates were incubated at 34°C for 20 minutes inside the luminometer. Luciferase assay solution was made by adding 450 *μ*L of NHS and 50 *μ*L of water to Sigma's ATP bioluminescence FLAAM assay reagent and incubating on ice for one hour. To assay for ATP release, 50 *μ*L of luciferase assay solution was injected into each well, and relative light units (RLUs) were recorded by the luminometer for a continuous 20 minutes. Data was analyzed as an integral over 20 minutes (sum RLUs). Following each assay, the wells were emptied and plates were snap-frozen. Protein assays were conducted on the assay plates by adding 200 *μ*L of the Pierce BCA Protein Assay Reagent A: Reagent B (1 : 50) mix (Pierce, Rockford, IL, USA) to the wells containing either cells or a BSA standard curve and incubated for 15 minutes at 37°C. Plates were then read using a BioRad Model 680 microplate reader (BioRad, Hercules, CA, USA) at 570 nm. Using the standard curve, the protein content of each well was estimated and used to normalized the integrated RLU measure. The control (DMSO) samples from each plate were randomized into two groups, “test” and “standard,” and the percent control was calculated as a percentage of the DMSO standard value. 

## 3. Results

### 3.1. Experimental Design

To further examine the mechanisms underlying OAG-stimulated ATP release in astrocytes, I used a live calcium imaging approach. Each imaging session consisted of one coverslip imaged in three separate time periods (*t*1, *t*2, and *t*3) in a contiguous experiment. T1 served as a baseline control measuring the response of the astrocytes to OAG. After a 15-minute wash period, *t*2 served as the experimental session where the response to OAG was examined in the presence of various drugs. After another 15-minute wash period, the *t*3 imaging period was used to examine recovery of the astrocyte response to OAG. 

### 3.2. OAG Induces Calcium Oscillations in Astrocytes

**Figure 1:**
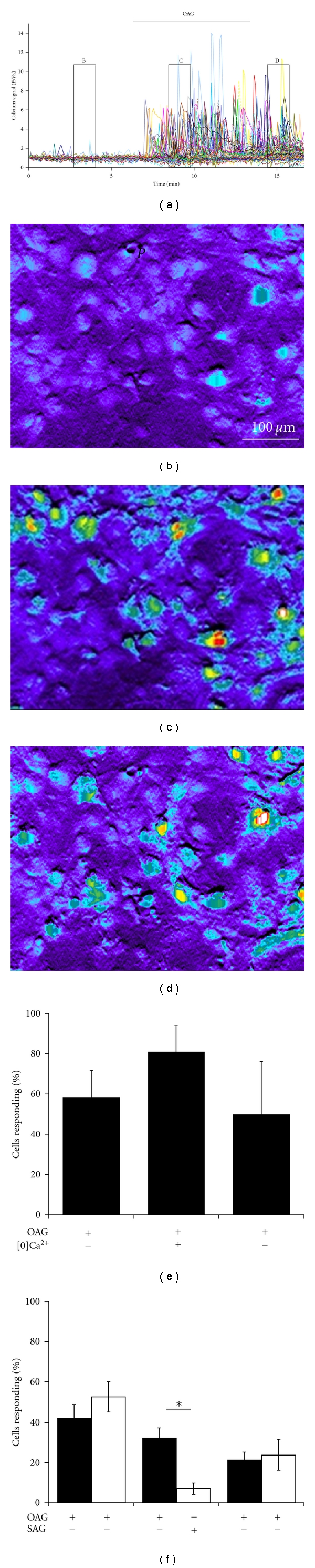
The diacylglycerol analogue 1-oleoyl-2-acyl-sn-glycerol (OAG) induces calcium oscillations in astrocytes that are not dependent on external calcium. (a) Cultured cortical astrocytes respond to 100 *μ*M OAG with robust calcium oscillations. (b) Astrocytes imaged in culture in a perfusion system have little background calcium activity as imaged with Fluo4. (c) The addition of 100 *μ*M OAG causes increases in the Fluo4 signal in discrete astrocytes but does not result in a calcium wave. (d) Astrocyte calcium oscillations continue for several minutes after the OAG-containing medium is washed away. (e) Calcium oscillation in response to 30 *μ*M OAG is not reduced in the presence of nominally (~60 nM) calcium-free external medium (*P* = 0.25, *n* = 5). (f) The astrocyte response to 30 *μ*M OAG is significantly greater than the response to the less cell-permeable DAG analogue 1-stearoyl-2-arachidonyl-sn-glycerol (SAG, 30 *μ*M) (*P* = 0.0002, control *n* = 14, SAG *n* = 6).

Similar to Hisatsune et al., I found that the application of OAG induced calcium oscillations in astrocytes (Figures 1(a), 1(b)–1(d)). This response was dose dependent, being more robust in the presence of 100 *μ*M OAG as compared to 30 *μ*M OAG (data not shown). Figures 1(b)–1(d) show 1-minute collapsed reconstructions of the cells represented by the traces in Figure 1(a). In Figures 1(b)–1(d), and Supplemental Figure 1 the calcium signal can be seen as discreet oscillations represented by the appearance of high Fluo4 signals (yellow-red) in noncontiguous cells in separate regions of the imaging field. The calcium response persists in nominally calcium-free external solution, indicating that internal stores are the source of the calcium oscillations (Figure 1(e)). The specificity of this response to OAG was insured by use of the DAG analogue 1-stearoyl-2-arachidonyl-sn-glycerol (SAG), which permeates the cell membrane much less readily than OAG, and therefore shows an attenuated response (Figure 1(f)). 

### 3.3. Astrocytes Release ATP in Response to OAG Application

**Figure 2:**
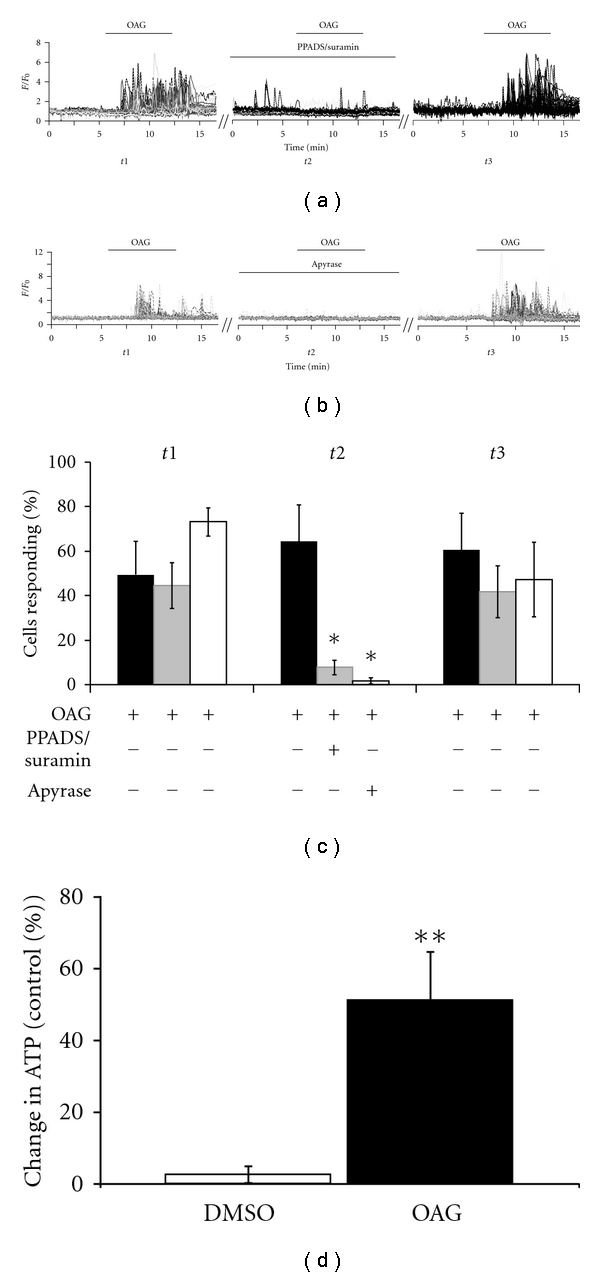
OAG-dependent calcium oscillations in cultured astrocytes are a result of ATP release. (a) During the first imaging period (*t*1) a baseline response to 100 *μ*M OAG is established in astrocyte cultures. Data are shown as individual cell traces of *F*/*F*_0_ (fold change) over time (minutes). Blocking astrocytic ATP receptors during the second imaging period (*t*2) with a combination of the P2 receptor inhibitors PPADS and Suramin greatly reduces the response to 100 *μ*M OAG. This blockade is reversible, after washout the response to 100 *μ*M is reestablished in the third imaging period (*t*3). Diagonal lines between imaging session indicate a wash period of 15 minutes occurring between imaging sessions. (b) Addition of the enzyme apyrase, which degrades ATP to ADP, and ADP to AMP, during *t*2 abolishes the astrocytic response to 100 *μ*M OAG. (c) Quantification demonstrates a significant decrease in the percentage of astrocytes responding with calcium oscillations to OAG stimulation with either the blockade of ATP receptors (PPADS/Suramin; control *n* = 5, PPADS/Suramin *n* = 8; *t*2  *P* = 0.02) or the degradation of extracellular ATP (Apyrase; control *n* = 5, apyrase *n* = 4; *t*2  *P* = 0.02). (d) ATP assays performed with the luciferin-luciferase reaction demonstrate the release of ATP from cultured astrocytes treated with OAG, compared to controls with DMSO added (DMSO *n* = 29, OAG = 27, *P* = 0.001).

I used the luciferase-luciferin reaction to examine OAG-stimulated astrocytes via luminometry. Astrocytes cultured in 96-well plates (*n* = one plate) released significantly more ATP in response to 30 *μ*M of OAG than an addition of an equal volume of NHS containing 0.05% DMSO (Figure 2(d)). These results indicate that stimulation of the DAG arm of the Gq pathway leads to ATP release in cultured astrocytes. 

### 3.4. Calcium Oscillations Are Induced by Activation of P2 Receptors by ATP

To test the hypothesis that the OAG-induced calcium oscillations were being induced in a paracrine manner by astrocytic ATP release, I used the ATP receptor blockers PPADS and Suramin in combination to block both P2Y and P2X receptors expressed in the cultured astrocytes. The astrocytic response to OAG was greatly diminished in the presence of ATP receptor antagonists (Figure 2(a)), indicating that the calcium response observed is downstream of ATP receptor activation. The enzyme apyrase degrades ATP to ADP, and ADP to AMP. In the presence of this enzyme, OAG application failed to induce calcium oscillations (Figure 2(b)). These results all appeared reversible upon the reapplication of OAG following washout, as summarized in Figure 2(c). Thus, the OAG-induced calcium oscillations result from the stimulation of astrocytic ATP receptors following astrocytic ATP release. 

### 3.5. Astrocytic ATP Release Is Not Mediated by DAG Metabolites

**Figure 3:**
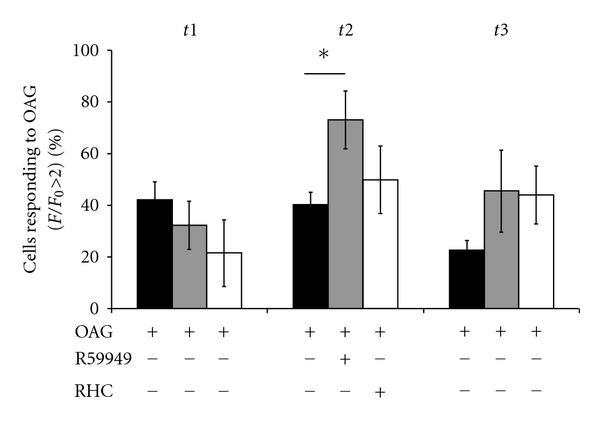
Blockade of DAG metabolites does not inhibit astrocytic ATP release. Inhibition of DAG kinase with 10 *μ*M R59945 causes an increase in astrocytic calcium oscillations in response to 30 *μ*M OAG (control *n* = 13,  *R*5  *n* = 5;  *P* = 0.016). Inhibition of DAG lipase with 50 *μ*M RHC80267 had no effect on astrocyte ATP release (control *n* = 6, RHC = 9;  *P* = 0.3).

The enzyme DAG kinase metabolizes DAG to phosphatidic acid (for review see [25]). DAG lipase metabolizes DAG to arachidonic acid [26]. Both of these small molecules have putative signaling capabilities [27]. However, OAG-induced calcium oscillations were not blocked by antagonists of either DAG lipase or DAG kinase. Upon observing the robust response to the 100 *μ*M concentration of OAG in these studies, I decided to use the more moderate dose of 30 *μ*M, which allowed us to see if there was either an incremental or detrimental effect of the pharmacological reagents tested. Treating cells with the DAG kinase inhibitor R59949 (10 *μ*M) enhances calcium oscillations caused by the lower dose (30 *μ*M) of OAG (Figure 3). Treatment with the DAG lipase inhibitor RHC80267 (50 *μ*M) had no effect. These data indicate that it is DAG itself, rather than one of its metabolites, which is primarily responsible for ATP release. 

### 3.6. OAG-Induced ATP Release Is Independent of PKC

Protein kinase C can be activated by DAG via its C1 domain and has been implicated in the modulation of exocytosis via the phosphorylation of proteins with roles in vesicle docking, priming, and fusion [28]. I used several pharmacological approaches to examine the role of PKC in OAG-induced astrocytic ATP release. Staurosporine is a general serine/threonine protein kinase inhibitor that blocks the ATP-binding domain of PKC as well as other kinases including protein kinase A and MAP kinase [29]. The bisindolylmaleimide GF 109203X (GFX) is a potent and selective inhibitor of protein kinase C that acts specifically at the ATP binding site [30]. Calphostin C interferes with the DAG-binding C1 domain of PKC, as well as of other proteins containing this domain [31].

**Figure 4:**
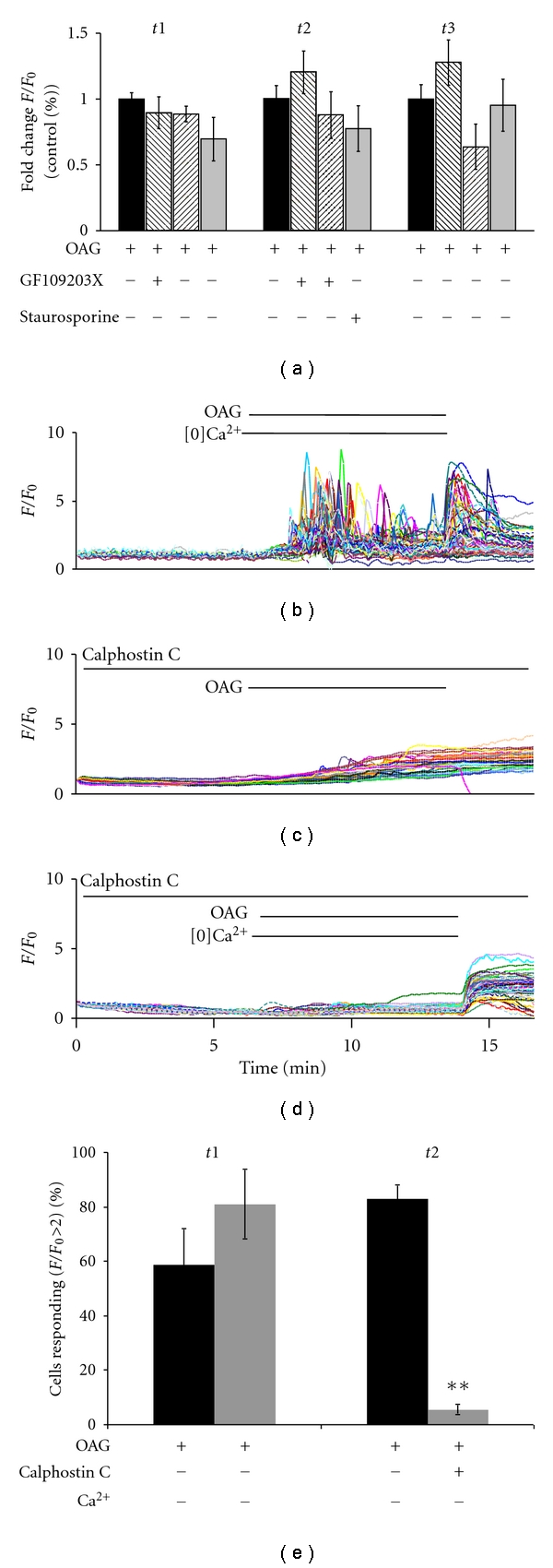
PKC activation is not necessary for OAG-induced release of ATP from astrocytes. (a) Adding 1 *μ*M GF 109203X (GFX) during the second imaging period does not reduce calcium oscillations (*P* = 0.3, control *n* = 8, GFX *n* = 5) in response to 100 *μ*M OAG. Pretreating astrocytes with 1 *μ*M GFX and including GFX during *t*1 and *t*2 does not reduce calcium oscillations induced by 100 *μ*M OAG (*P* = 0.14, control *n* = 8, GFX = 5), although there is a significant (*P* = 0.03) increase in calcium oscillations after washout in this GFX condition. The serine/threonine kinase inhibitor staurosporine does not have an effect on calcium oscillations induced by 100 *μ*M OAG when added during *t*2 (*P* = 0.36, control *n* = 7, staurosporine *n* = 4). (b) Astrocytes display robust OAG-induced calcium oscillations in nominally calcium-free external solution. (c) Incubation with 100 nM Calphostin C during a 15-minute light activation period followed by incubation during *t*2 abolished calcium oscillation and induced a slow increase in Fluo4 signal during the OAG incubation. (d) The slow increase in Fluo4 signal is abolished in the absence of external calcium and oscillations are absent in the presence of 100 nM Calphostin C and 30 *μ*M OAG. (e) Calcium oscillations in nominally calcium-free medium induced by 30 *μ*M OAG are significantly reduced by the addition of 100 nM Calphostin C (*P* = 0.004, control *n* = 5, calphostin C *n* = 4). As calphostin C is irreversible, there is no washout period (*t*3) for (e).

Adding GFX at time 2 had no effect on the response of astrocytes to OAG (Figure 4(a)). However, a recent report found that previous activation of PKC with a phorbol ester may render it active even in the presence of GFX [32]. When I pretreated cells with GFX, before OAG application, there was still no effect upon OAG-induced calcium oscillations (Figure 4(a)). Staurosporine application also had no response. During calphostin C application, there was a gradual increase in the calcium signal (Figure 4(c)). This was abolished by incubation in nominally calcium-free external NHS (Figure 4(d)), indicating that the signal was a result of an influx of external calcium, perhaps via TRPC channels, which can be activated by DAG but do not contain C1 domains [33, 34]. Cultured astrocytes respond to OAG in calcium-free external NHS with oscillations (Figures 1(b) and 4(b)). However, treatment with calphostin C abolished this response (Figure 4(d); summarized in Figure 4(e)). Thus, specific inhibition of PKC did not affect astrocytic ATP release, but inhibition of proteins containing C1 DAG-binding domains abolished the response to OAG. 

### 3.7. Mechanisms of ATP Release from Astrocytes

**Figure 5:**
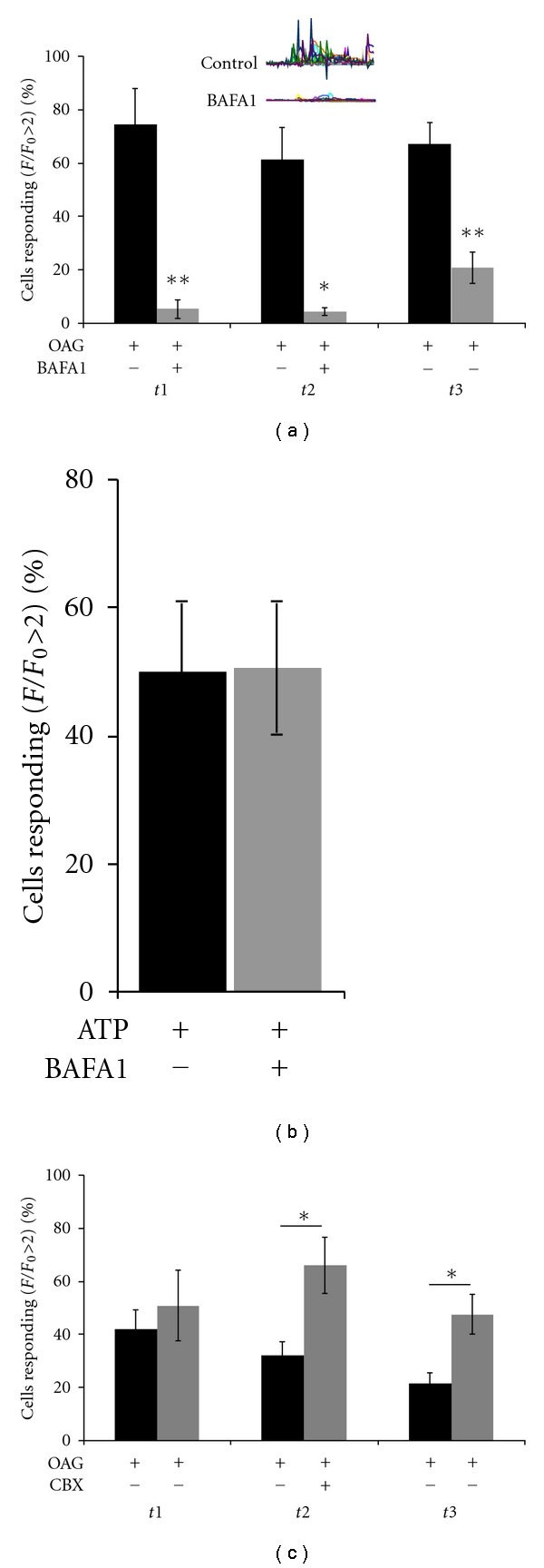
OAG-induced ATP release from astrocytes appears to be vesicular. (a) Incubation of cultured astrocytes with 100 nM Bafilomycin A1, an inhibitor of the vesicular H^+^  ATPases that set up the proton gradients necessary for vesicle filling, greatly reduced OAG-induced astrocytic ATP release (bafilomycin *n* = 8, control *n* = 5;  *t*1  *P* = 0.01,  *t*2  *P* = 0.02,  *t*3  *n* = 0.004). This effect was partially washed out after 15 minutes (*t*2 bafilomycin versus *t*3 wash *P* = 0.024). (b) Bafilomycin treatment did not affect the function of astrocytic ATP receptors, as the calcium response to 1 *μ*M ATP was unaffected by bafilomycin treatment (bafilomycin *n* = 5, control *n* = 5, *P* = 0.9). (c) The gap junction and hemichannel blocker carbenoxolone (CBX), at 50 *μ*M, resulted in an increase in astrocytic ATP release (control *n* = 14, CBX *n* = 5;  *t*2  *P* = 0.03, *t*3  *P* = 0.02).

A number of mechanisms have been suggested for the release of ATP from astrocytes. Two of the most likely under normal physiologic conditions are the release of ATP via vesicular exocytosis [1, 35–37] and regulated release via hemichannels [38, 39]. Bafilomycin is an inhibitor of the vesicular H^+^  ATPases that set up the proton gradients necessary for vesicle filling [40] and is a drug commonly used to probe exocytotic mechanisms. Preincubation with bafilomycin A1 to deplete vesicles and prevent vesicle filling greatly diminished OAG-induced astrocytic ATP release (Figure 5(a)). The calcium response to 1 *μ*M ATP was not changed by bafilomycin treatment, indicating that the astrocytic ATP receptors were not affected by this treatment (Figure 5(b)). This indicates that vesicular release is a likely mechanism underlying the OAG-induced ATP release from cultured astrocytes. Hemichannels can be selectively blocked by the glycyrrhetinic acid derivative carbenoxolone [41–43]. Incubation with 50 *μ*M carbenoxolone did not block OAG-induced ATP release in cultured astrocytes (Figure 5(c)). Indeed, there was a significant increase in OAG-induced ATP release after incubation with this hemi- and gap-junction blocker. 

**Figure 6:**
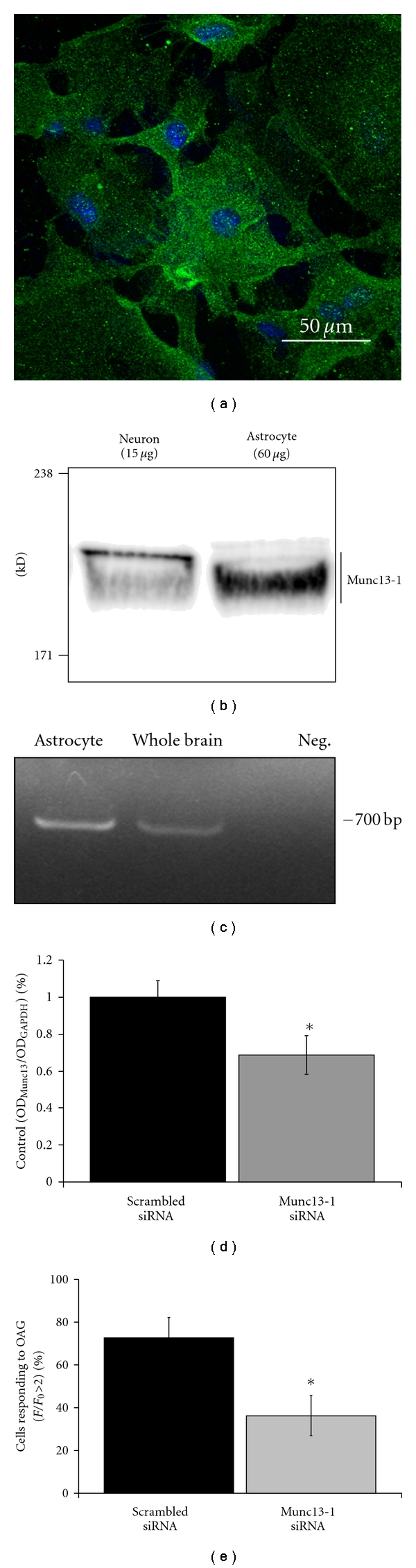
The Munc13-1 protein is expressed in cultured astrocytes and reduction of Munc13-1 expression reduces astrocytic ATP release. (a) Immunocytochemistry for Munc13-1 in cultured astrocytes demonstrates expression of Munc13-1 in these cells: (green: Munc13-1, blue: DAPI). (b) Western blotting for Munc13-1 demonstrates a band at the expected size (~200 kD). Gel lanes were loaded with 60 *μ*g of cultured mouse astrocyte protein or 15 *μ*g of protein extracted from cultured mouse cortical neurons. (c) RT-PCR with primers designed against Munc13-1 of whole mouse brain mRNA and mRNA extracted from cultured astrocytes demonstrated a band for Munc13-1 at the expected size. Sequencing of these bands confirmed the presence of Munc13-1 in cultured astrocytes. (d) siRNAs designed against mouse Munc13-1 reduce the expression of Munc13-1 in transfected mouse astrocytes as measured by Western blot. Optical density of the Munc13-1 signal was standardized against the housekeeping protein GAPDH (scrambled siRNA *n* = 4, Munc13-1 siRNA = 4, *P* = 0.05). (e) The response of cultured astrocytes to OAG during one imaging session was measured for cultured astrocytes transfected with either scrambled control siRNAs or siRNAs directed against the Munc13-1 mRNA. The percentage of cells responding to OAG was significantly reduced after transfection with Munc13-1 siRNA (scrambled siRNA *n* = 6, Munc13-1 siRNA = 9; *P* = 0.017).

The inhibition of calcium oscillations by calphostin C suggests that astrocytic ATP release is mediated via a DAG-binding protein other than PKC. The Munc13-1 protein has been shown to be crucial for transmitter release from cell types including chromaffin cells and neurons [44–46]. Munc13-1 has been shown to potentiate neurotransmitter release via both PKC-dependent and independent pathways following DAG activation [32]. I show that Munc13-1 mRNA and protein are expressed in cultured cortical astrocytes (Figures 6(a)–6(c)). siRNas designed against Munc13-1 were able to reduce Munc13-1 protein content, as measured via Western blot in cultured astrocytes, by about 30% (Figure 6(d)). This modest siRNA-mediated reduction of Munc13-1 likely reflects its rapid turnover rate [47]. The reduction of Munc13-1 in astrocytes significantly reduces OAG-induced astrocytic ATP release (Figure 6(e)) implicating this protein in exocytotic release of ATP from astrocytes. 

## 4. Discussion

The current work seeks to elucidate the pathway linking the activation of Gq-coupled receptors to gliotransmission in the astrocytes. I have shown, using in vitro live imaging, that stimulation of the DAG pathway with the DAG analogue, OAG, results in ATP release that appears to be both PKC independent and vesicular. Furthermore, I have shown evidence implicating the Munc13-1 exocytotic protein in this novel mechanism. 

Following my initial observation that the application of OAG led to ATP release from astrocytes, as measured directly via luminometry, I primarily used calcium imaging as an indirect measurement of astrocytic ATP release. This has proven to be a reliable and robust indicator of ATP release in these cells (for review see [48]), and pharmacological reagents can be easily washed via a tightly controlled perfusion system. Two concentrations of OAG reliably induced calcium oscillations in cultured astrocytes. These oscillations were observed in individual cells in distinct areas of the culture; calcium waves were not observed upon OAG application (Supplemental Figure 1). Calcium waves in cultured astrocytes have been attributed to ATP released acting upon the ATP receptors of neighboring astrocytes [49]. Because the ATP released in these studies did not trigger calcium waves in the confluent cultures I imaged, I can assume that the amounts released were very small, and fed back onto ATP receptors no further distant than the immediate cell concerned. 

Previous work confirmed that OAG-induced astrocyte calcium oscillations are PLC dependent [19]. The use of PPADS combined with suramin to block P2 receptors allowed us to observe that the OAG-induced oscillations were dependent upon functional ATP receptors. Combined with the use of apyrase, which metabolizes ATP to ADP and ADP to AMP, these data indicate that the OAG-induced calcium oscillations are being mediated by ATP acting upon P2 receptors to mobilize calcium from internal stores. The calcium responsible for the Fluo4 fluorescence was not coming from the external solution, as OAG-induced oscillations persisted in the absence of external calcium. 

There are a number of pathways downstream of the conversion of PLC into DAG and IP_3_. Following the production of DAG, it is possible for this molecule to bind to the C1 domains of PKC [50], Munc13, DAG Kinase, chimaerin, and Ras GRPs (for review see [51]). DAG can also bind proteins, such as DAG lipase and membrane TRP channels [34], which do not contain C1 domains. DAG kinase and DAG lipase metabolize DAG to phosphatidic acid and arachidonic acid, respectively [25, 26]. Both of these metabolites have signaling capabilities [27]. The inhibition of either enzyme did not perturb the response of the astrocytes to OAG in these studies. Thus, neither arachidonic acid nor phosphatidic acid is necessary for the OAG-induced ATP release. OAG is likewise a substrate for both of these enzymes; therefore the lack of effect is not from a lack of these metabolites. The increase of the OAG-induced ATP release induced via inhibition of DAG kinase with R59949 suggests that DAG kinase is the more predominant metabolic enzyme for DAG in cultured cortical astrocytes.

DAG activation of PKC has long been known to potentiate synaptic function [52–55]. The bisindolylmaleimide GF 109203X (GFX) is a potent and selective inhibitor of both classical and novel PKC isoforms [30, 56]. In the current study, neither pretreatment nor regular application of GFX prevented OAG-induced ATP release. Application of the C1 domain inhibitor calphostin C in normal external medium caused an influx of external calcium. This could be due to the actions of OAG and endogenous DAG upon astrocytic TRPC channels, which bind DAG but do not contain a C1 domain [33]. In nominally calcium-free external medium, the calcium influx is prevented. Although OAG normally stimulates ATP release in the presence of calcium-free external medium, this OAG-dependent ATP release is blocked by calphostin C. DAG potentiation of synaptic vesicle release has also been suggested to be, in some cases, PKC independent and Munc13-1 dependent [57]. Munc13-1 potentiation of transmitter release in neurons may also rely upon previous phosphorylation of the Munc18 protein by PKC [32]. I show here that DAG appears to potentiate ATP release in astrocytes independent of PKC. 

The bafilomycin data presented in this study support a vesicular release of ATP downstream of OAG stimulation in cultured astrocytes. However, astrocytes have also been purported to release ATP via connexin or pannexin hemichannels [38, 58, 59]. After blocking astrocytic hemichannels with carbenoxolone, I found that the OAG-induced ATP release from cultured astrocytes was not diminished but instead was significantly enhanced. While this result argues against hemichannel ATP release being induced by OAG, the augmentation of the effect with carbenoxolone is intriguing. Perhaps, as carbenoxolone also blocks intercellular gap junctions [41], IP_3_ is unable to diffuse to coupled cells [60] and thus more calcium is released from intracellular stores. Alternatively, the inhibition of intercellular gap junctions may also prevent the diffusion of cytosolic ATP between confluent astrocytes [61], increasing the availability of ATP for uptake into vesicles.

The role of Rho GTPases in nonexocytotic astrocytic ATP release has been examined [62]. Utilizing an astrocytoma cell line, the authors implicated Rho GTPases as an effector of hemichannel ATP release downstream of Gq activation. While this is an intriguing possibility, my studies knocking down Munc13-1 expression in primary confluent astrocytes direct attention towards that protein as being involved in astrocytic ATP release. The significant reduction in ATP release I find, upon a relatively modest siRNA-mediated reduction in Munc13-1 expression, implies that ATP release may be tightly controlled by Munc13-1 in astrocytes. It has previously been shown that astrocytic ATP release is impaired by the disruption of the SNARE exocytotic machinery [1], which interacts with the MUN domain of Munc13-1 in neurons [63]. The data presented in this work concur with a vesicular release of ATP in astrocytes and provide evidence for a DAG/Munc13 pathway linking Gq signaling in astrocytes with ATP gliotransmission. 

## Supplementary Material

Figure 1: Cultured astrocytes response to OAG stimulation with discrete calcium oscillations. Astrocytes loaded with the calcium indicator Fluo4 show little activity at baseline in a continuous perfusion of NHS. Upon the application of OAG (green box), discreet oscillations can be observed as uncoordinated increases in Fluo4 signal in different regions of the viewing field.
